# Stage-Specific Effects of Population Density on the Development and Fertility of the Western Tarnished Plant Bug, *Lygus hesperus*

**DOI:** 10.1673/031.010.4901

**Published:** 2010-05-17

**Authors:** Colin S. Brent

**Affiliations:** USDA Arid Land Agricultural Research Center, 21881 N. Cardon Lane, Maricopa, AZ 85238, USA

**Keywords:** environmental stress, fecundity, fluctuating asymmetry, Miridae, mortality rate

## Abstract

The western tarnished plant bug *Lygus hesperus* Knight (Heteroptera: Miridae), a major pest of cotton and other key economic crops, was tested for its sensitivity to population density during nymph and adult stages. Nymphs reared to adulthood under increasing densities in laboratory conditions exhibited incremental delays in maturation, heightened mortality rates, and reductions in body mass and various size parameters. In contrast, gonadal activity in both males and females rose with initial density increases. Supplemental nutrients provided to the nymphs failed to offset the negative effects of high density, suggesting that contact frequency, rather than resource partitioning, may be the primary stress. Unlike nymphs, newly eclosed adults exposed to increasing population densities did not suffer negative physiological effects; body mass, mortality rates and patterns of ovipositional activity were unchanged. Collectively, these results indicate that population density can dramatically influence *Lygus* development, but the specific effects are stage-dependent.

## Introduction

The western tarnished plant bug, *Lygus hesperus* Knight (Heteroptera: Miridae) is a pest of numerous fiber, fruit, seed and vegetable crops (Jackson et al. 1995). Despite their economic importance, relatively little is known about the specific environmental factors influencing their development and fecundity. For many higher organisms, a key influential factor is the density of the population in which they live. Depending on environmental conditions and host plant quality, field population densities of *L. hesperus* can fluctuate widely (i.e. Bancroft 2005; Carrière et al. 2006; Demirel and Cranshaw 2006), but even under ideal conditions they seldom achieve the densities found in experimental laboratory colonies. Insects reared at such artificially high densities often show evidence of abnormal or retarded development, reduced fertility, and exacerbated mortality rates (reviewed in Peters and Barbosa 1977; Clarke and McKenzie 1992; Hoffman and Woods 2001). These negative effects are often a consequence of increased intraspecific competition for limited nutritive resources, but even an increased contact rate from living in close proximity can influence development and behavior in insects, as observed in *Schistocerca gregaria* (reviewed in Simpson et al. 1999). Identifying the developmental and behavioral responses to such potential stressors can facilitate the design of rearing environments that avoid potential confounding effects in tests using laboratory populations.

The responses to such environmental stressors can vary not only by species and severity of the stimuli, but they can also be influenced by the developmental status of an insect. The effects of stress tend to be greater and more persistent in immature stages than in adults (reviewed in Peters and Barbosa 1977), although there is evidence that, for some species, negative consequences do not always translate into adulthood (see Campero et al. 2008). Fully mature adults exposed to poor environmental conditions tend to have short-term and reversible responses that change as their environment fluctuates. Adjustments in gamete production, mating behavior, and dispersive tendencies are common (Peters and Barbosa 1977). Adults of several Mirid species have been observed exhibiting such adaptive responses. For instance, *Lygus lineolaris* appears to adjust oviposition and migratory habits to match host density under field conditions (Rhainds and English-Loeb 2003). Similarly, increasing population density reduces female fertility in *Dicyphus tamaninii* (Agustı' 1998) and reduces male mating behavior in *Macrolophus caliginosus* (Castaňé et al. 2007).

This study was designed to determine if the high population densities used in laboratory rearing conditions negatively affect the rate and extent of nymph maturation and the oviposition rates of adults. Mixed-sex groups of *L. hesperus* were reared together at three different population densities for each developmental stage. The effect of nutrient access was also tested in nymphs. Nymph development and reproductive maturation were assessed by a series of morphological measures, including a composite measure of fluctuating asymmetry. Mortality rates were tracked for both adults and nymphs.

## Materials and Methods

### Insects

The *L. hesperus* used in this study were obtained from a laboratory-reared stock colony maintained at the US Arid Land Agricultural Research Center (Maricopa, AZ, USA). The individuals in this colony are periodically outbred with locally-caught conspecifics. The stock insects were given unrestricted access to a supply of green beans and an artificial diet mix (Debolt 1982) packaged in Parafilm (Patana 1982). These nutritive sources were replenished as needed. Similar Parafilm coated packets of an agar solution (15 g/liter), were provided to the females as a site for ovipositing, and are hereafter referred to as “egg packets.” Insects were reared at 25° C, 20% relative humidity, under a 14: 10 L:D photoperiod.

### Nymph Population Density

To synchronize the age of nymphs used in experiments, groups of mixed-sex *L. hesperus* were allowed to oviposit for 4 h, and the fresh egg packets were collected. Nymphs were collected after 2–3 d. Post oviposition age was noted, and the nymphs were transferred into a 355 ml rearing cup (Huhtamaki, www.huhtamaki.com). Because, under natural conditions, the density of each developmental stage could affect development (Peters and Barbosa 1977), and because both the density and age composition of a localized population can change over time (Schotzko and O'Keefe 1989b), single age cohorts were used to mitigate possible confounding influences. Each rearing cup contained two 10 × 10 cm wax paper sheets crumpled to provide additional walking surface, approximately 12 g of fresh green beans, a 12 g artificial diet paraffin packet, and a wire mesh screen to support the food. Diet was replaced every 48 h to ensure freshness. The cups were made of waxed chipboard and were covered with a nylon mesh to ensure adequate air circulation and light exposure. To test for density effects, cups contained both sexes of nymphs in one of three different population sizes (20, 100, or 500). For each density, 14, 10, and 12 cups were prepared, respectively. Sixteen additional cups with 500 nymphs and twice the normal diet (designated 500DD hereafter) were prepared to determine if any negative impact associated with the increased population density resulted from increased competition for limited nutrients.

The number of living nymphs was determined every 24 h to track both mortality rates and the number that had molted into adults. After 50% of the survivors reached adulthood, all adults were preserved in 50% ethanol for a minimum of one week. Ten adult males and 10 females were randomly selected from each cup and dissected to assess their developmental state. In cups with starting populations of 20, data were collected from all individuals still living at the end of the sample period.

Several physiological parameters were measured. First, individual wet body mass was measured (Sartorius TE153S), after excess ethanol solution was dried from the external surface. Several length measurements were taken using a dissecting scope (Zeiss Stemi SV6) equipped with an ocular micrometer and calibrated to a stage micrometer. Length and width were measured for both forewings. Wing length was calculated from the point of attachment to the thorax to the posterior tip, and width was the perpendicular distance from the angle of the wing where its meets the posterior tip of the scutellum to the opposite side of the wing. The lengths of the second segment from the proximal end of both antennae were also measured. For many of the individuals sampled, the antennae were missing one or more distal segments, but the second segment was almost always present and provided an indicator of overall antenna length. Lastly, the width of the pronotum was determined at its widest point, as an indicator of overall body size.

Because environmental stress can also contribute to malformations resulting in non-symmetrical development (Allendorf and Leary 1986; Palmer and Strobeck 1986; Clarke and McKenzie 1992; Leung and Forbes 1996), asymmetry scores can potentially reveal more than simple length measures. Individual fluctuating asymmetry values were calculated by measuring the deviation from perfect bilateral symmetry for each of the paired trait measures (wing length and width, antennal segment length). Within each trait and across all groups, the absolute values of these deviations were ranked to ensure standardization. These ranked fluctuating asymmetry values were summed for each individual to create a statistical composite score. The composite fluctuating asymmetry scores were then used for intergroup comparisons to determine if increasing population size can influence symmetry (Leung et al. 2000).

The final trait assessed was degree of gonadal development and activity. *Lygus* oocyte development goes through three distinct phases (pre-vitellogenic, vitellogenic, choriogenic; Ma and Ramaswamy 1987). “Based on this pattern, a four stage scale was used to rate ovarian activity: 0 = previtellogenic oocytes only; 1 = one 0 = no vitellogenic oocytes; 1 = one or more slightly vitellogenic oocytes; 2 = one or more highly vitellogenic oocytes; 3 = vitellogenic oocytes with a pigmented operculum. For males, the length, width, and height of each testis was measured and used to calculate volume. One testis per male was then homogenized in 20 µl of distilled water. A 10 µl aliquot of the homogenate was placed on a Spencer Brightline hemocytometer and spermatozoa were counted under a stereomicroscope to calculate sperm concentration per testis. Because the testes of *L. hesperus* do not store sperm (Strong et al. 1970), these measures provided a rough estimate of the relative rates of spermatogenesis among males.

### Adult Population Density

The effect of population density on the body mass and oviposition rates of adults was separately assessed by maintaining groups of adults of known age at increasing densities (20, 60, and 100 individuals). For each density, equal numbers of newly molted female and male adults were placed in 15 – 355 ml rearing cups. These individuals spent their nymph stage in 1890 ml cups in groups of 100, which is roughly equivalent to the population density of 20 nymphs in 355 ml cups (0.053 vs. 0.056 nymphs/ml, respectively). This was done to ensure that any observable effects on adult mass and egg production were due solely to the conditions experienced as adults and not as residual effects of the nymph rearing environment. Adults were reared under the same conditions as outlined above for nymphs, with the inclusion of an egg packet. The number of live adults and oviposited eggs was censused every 24 h after cup initiation. After 10 d, a sufficient amount of time for most adults to have fully mature and active gonads, 10 individuals of each sex were randomly selected from each cup and preserved in 50% ethanol. Individual body masses were determined and female ovarian activity was assessed as described for nymphal studies.

### Statistical Analysis

Initial comparisons between multiple groups were conducted using ANOVA, correcting for multiple paired comparisons using the Holm-Sidak method. In cases where the data were non-normally distributed, a Mann-Whitney rank sum test was used for paired comparisons, and for multiple comparisons a Kruskal-Wallis ANOVA was used, corrected by Dunn's method. A Spearman rank order correlation was used to determine the associations between testis volume and sperm number. All analyses were conducted using Sigmaplot 11.0 (Systat Software).

## Results

### Nymph Population Density

Increasing the population density had a negative effect on every measured development trait. Some of the most pronounced effects were observed in the groups of 500 that had also received twice the normal amount of food (500DD). Although the increased number of green beans and the second artificial diet packet supplied additional nutrients, the added food effectively decreased the available volume within the cups. These data suggest that this produced a further increase in the population density.

**Figure 1.**
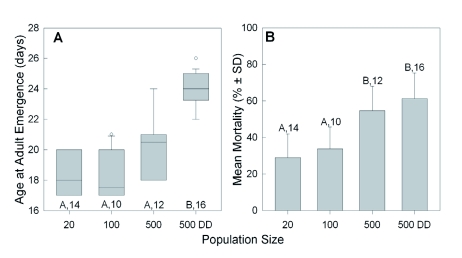
Effect of increasing population size and food availability (DD = doubled diet) in fixed volume cups on (A) the median number of days after being oviposited that 50% of co-maturing *Lygus* *hesperus* individuals have molted into adults; and (B) the mean (± S.D.) mortality rate at this time. The boxplots shown in (A) also indicate the interquartile range, the largest non-outlier value (whiskers), and the outliers (circles). Significant differences among groups are indicated by different letters (p < 0.05, Dunn's or Holm-Sidak multiple comparison test after ANOVA for A & B, respectively). Sample sizes are given. High quality figures are available online.

Raising the population density in the rearing cups significantly increased the median amount of time it took for nymphs to initiate the adult molt (Kruskal-Wallis ANOVA, H = 33.5, df = 3, p < 0.001), from 18 d in groups of 20 to a high of 24 d in the 500DD groups (Figure 1A). The mean mortality rates within the cups also increased significantly (Figure 1B; ANOVA, F = 21.6, df = 3, p < 0.001), more than doubling between those two groups, from 28.9% to 61.3%.

**Figure 2.**
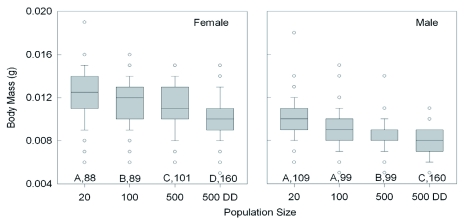
Effect of increasing population size and food availability (DD = doubled diet) on the median body mass attained by female and male *Lygus* *hesperus* that had recently molted into adults. The boxplots also indicate the interquartile range, the largest non-outlier value (whiskers), and the outliers (circles). Significant differences among groups are indicated by different letters (p < 0.05, Dunn's multiple comparison test after ANOVA). Sample sizes are given. For each test condition, females massed significantly more than males (Mann-Whitney test, p < 0.01). High quality figures are available online.

**Figure 3.**
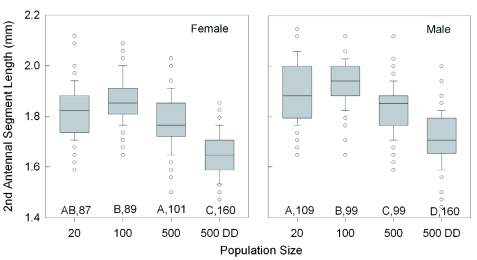
Effect of increasing population size and food availability (DD = doubled diet) on the median wing length of female and male *Lygus hesperus* that had recently molted into adults. The boxplots also indicate the interquartile range, the largest non-outlier value (whiskers), and the outliers (circles). Significant differences among groups are indicated by different letters (p < 0.05, Dunn's multiple comparison test after ANOVA). Sample sizes are given. For each test condition, females had significantly longer wings than males (Mann-Whitney test, p < 0.01). High quality figures are available online.

**Figure 4.**
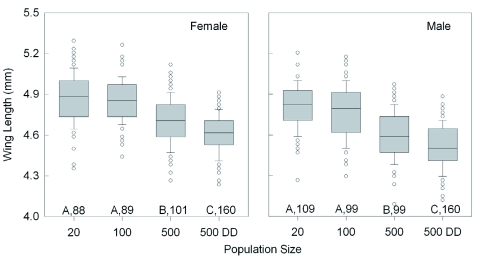
Effect of increasing population size and food availability (DD = doubled diet) on the median length of the second antennal segment of female and male *Lygus hesperus* that had recently molted into adults. The boxplots also indicate the interquartile range, the largest non-outlier value (whiskers), and the outliers (circles). Significant differences among groups are indicated by different letters (p < 0.05, Dunn's multiple comparison test after ANOVA). Sample sizes are given. For each test condition, females had significantly shorter segments than males (Mann-Whitney test, p < 0.01). High quality figures are available online.

**Figure 5.**
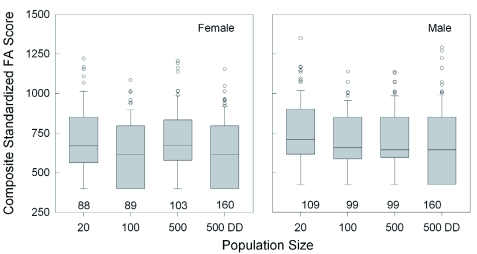
Effect of increasing population size and food availability (DD = doubled diet) on the fluctuating asymmetry of wing and antenna development. Left/right differences were standardized by ranking, then combining, them to produce a composite measure for analysis. The boxplots also indicate the interquartile range, the largest non-outlier value (whiskers), and the outliers (circles). There were no significant differences among groups or between sexes. Sample sizes are given. High quality figures are available online.

Increasing density also caused incremental size reductions in all of the external body population density (20 vs 500DD), adult body mass (Figure 2) declined 19.4% in females (t = 8.7, p < 0.001; Holm-Sidak) and 19.6% in males (Q = 9.4, p < 0.05; Dunn's Method). Across all of the rearing conditions, adult female body mass was significantly greater than that of the males (Mann-Whitney rank sum test; T_(438,467)_ = 25,8584.5; p < 0.001). Comparing the 20 and 500DD groups, wing length (Figure 3) declined by 5.2% in females (Q = 9.8, p < 0.05; Dunn's Method) and 6.1% in males (Q = 11.2, p < 0.05) with increasing density. The wings of females were consistently longer than those of males (Mann-Whitney rank sum test; T_(438,467)_ = 21,9163.0; p < 0.001). The results for wing width were nearly identical to those for wing length, with a decline of 3.2% in females and 3.4%) in males between the two density extremes. The length of the second antennal segment (Figure 4) also declined with increasing density, by 9.4% in females (Q = 10.55, p < 0.05; Dunn's Method) and 8.9% in males (t = 13.6, p < 0.001; Holm-Sidak). Unlike body mass and wing length, the females had smaller antennal segments than the males across all groups (Mann-Whitney rank sum test; T_(437,467)_ = 16,5750.5; p < 0.001). Finally, pronotum width (Figure 5) decreased incrementally across the groups, with a difference of 4.7% in females (Q = 6.3, p < 0.05; Dunn's Method) and 5.5% in males (Q = 8.7, p < 0.05; Dunn's Method). Female pronotums were significantly larger than those of males for all population conditions (Mann-Whitney rank sum test; T_(438,467)_ = 24,5358.0; p < 0.001).

**Figure 6.**
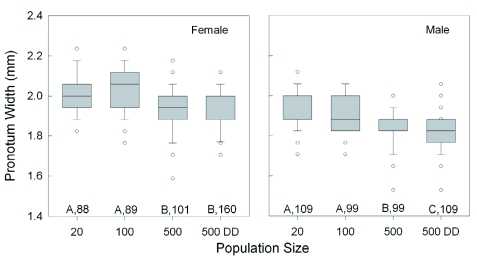
Effect of increasing population size and food availability (DD = doubled diet) on the median pronotum width of females and males that had recently molted into adults. The boxplots also indicate the interquartile range, the largest non-outlier value (whiskers), and the outliers (circles). Significant differences among groups are indicated by different letters (p < 0.05, Dunn's multiple comparison test after ANOVA). Sample sizes are given. For each test condition, females had significantly wider pronotums than males (Mann-Whitney test, p < 0.01). High quality figures are available online.

Despite the negative developmental effects of increasing density, very little asymmetry was observed in the formation of the wings and antennae. The mean trait variation across all population sizes and sexes was low (1.22% ± 0.03%), *n* = 3293). There were no differences among individuals from different rearing environments (Figure 6) for both females (Kruskal-Wallis ANOVA, H = 7.7, df = 3, p = 0.054), and males (Kruskal-Wallis ANOVA, H = 3.3, df = 3, p = 0.35). There were also no differences between the sexes (Mann-Whitney rank sum test; T_(433,463)_ = 19,3297.0; p = 0.8).

**Figure 7.**
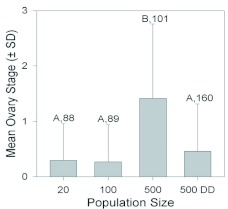
The mean (± S.D.) ovarian stage (0 = no vitellogenic oocytes; 1 = one or more slightly vitellogenic oocytes; 2 = one or more highly vitellogenic oocytes; 3 = vitellogenic oocytes with a pigmented operculum) of females that had recently molted into adults after being reared under different population sizes or dietary conditions (DD = doubled diet). Significant differences among groups are indicated by different letters (p < 0.05, Holm-Sidak multiple comparison test after ANOVA). Sample sizes are given. High quality figures are available online.

**Figure 8.**
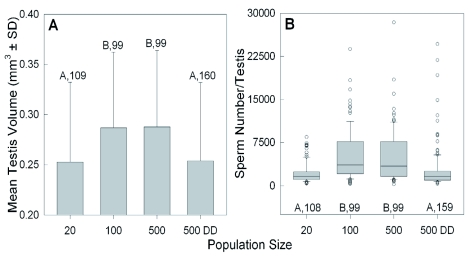
Effect of increasing population size and food availability (DD = doubled diet) on (A) the mean (± S.D.) testis volume; and (B) the median sperm number per testis in adult males sampled after 50% had eclosed. The boxplots shown in (B) also indicate the interquartile range, the largest non-outlier value (whiskers), and the outliers (circles). Significant differences among groups are indicated by different letters (p < 0.05, Holm-Sidak or Dunn's multiple comparison test after ANOVA for A & B, (respectively). Sample sizes are given. High quality figures are available online.

In addition to affecting external morphology, the increasing population sizes also influenced the gonadal development of both female and male nymphs, although the changes induced were neither consistently negative nor incremental. Females reared in groups of 500 achieved a significantly higher ovarian stage than those from the other groups (Figure 7; H = 73.9, df = 3, p < 0.001; Kruskal-Wallis ANOVA). Although those in the 500DD group also achieved a slight increase in oocyte production, the difference from the females in groups of 20 and 100 was only suggestive (Kruskal-Wallis ANOVA, H = 5.8, df = 2, p = 0.054). For males, testicular development increased significantly when reared in groups of 100 or 500, compared to those in groups of 20 or 500DD. There were significant changes in both testis volume (Figure 8A; ANOVA, F = 7.3, df = 3, p < 0.001) and sperm number (Figure 8B; Kruskal-Wallis ANOVA, H = 75.5, df = 3, p < 0.001). However, there was only a weak correlation between testis size and sperm amount (Spearman Rank Order, r = 0.25, p < 0.001, *n* = 465).

### Adult Population Density

**Figure 9.**
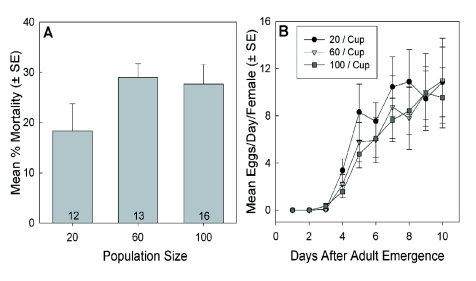
Effect of increasing population size on the mean (± S.E.) (A) percent mortality ten days after adult eclosion; and (B) individual oviposition rates during each of the first 10 days post-eclosion. There were no significant differences among treatments for either measure. Sample sizes given in A also apply to B. High quality figures are available online.

As was observed with nymphs, increasing population size among adults from 20 to 100 individuals caused an insignificant increase in the mortality rate recorded after 10 days of being grouped together (Figure 9A; ANOVA, F = 1.844, p = 0.172). In contrast to the nymphs, adult body mass after 10 days was unaffected by increasing population for both females (Kruskal-Wallis ANOVA, H = 1.2, df = 2, p = 0.54) and males (Kruskal-Wallis ANOVA, H = 0.8, df = 2, p = 0.67), but the females (overall median = 0.012 g) significantly outweighed the males (overall median = 0.008 g; Mann-Whitney rank sum test; T(158,161)= 120.0, p < 0.001).

The relative ovarian activity in 10 d old adults did not differ between groups (median = 4 in all; Kruskal-Wallis ANOVA, H = 1.0, df = 2, p = 0.602), nor did the rate at which females oviposited eggs (Figure 9B; Kruskal-Wallis ANOVA, H = 0.0976, df = 2, p = 0.95). The pace of egg deposition followed the same pattern for all population densities, with the first eggs appearing after 4 d and the oviposition rates plateauing after 8 d.

## Discussion

The results provide evidence that high population density can have a considerable negative impact on the development and mortality of *L. hesperus.* In nymphs, the effects of overcrowding were apparent for almost every physiological measure, reducing body mass and size and increasing mortality. In contrast, the only substantial effect on adults was for mortality rate. This suggests that the sensitivity to this stressor may decline as a *Lygus* matures or that different coping mechanisms are utilized at each stage.

The strong response of the nymphs to overcrowding has been observed in immature stages of other insect species and, in many cases, has been attributed to competition for limited nutrients (e.g. Averill and Prokopy 1987; Agnew et al. 2000; Reiskind et al. 2004). A restricted diet can directly impact mass gain and may also influence the timing and pattern of gene expression, resulting in delayed maturation and reduced body size. However, each trait may be affected independently by overcrowding rather than by a linked response. For example, differences in body mass arising from competition for nutrients has been found to have little direct consequence on other developmental traits in some insect species (Peters and Barbosa 1977).

A restricted diet isn't the only possible stress factor to which the *Lygus* nymphs were responding. Given that food supplies were replaced every other day and that doubling the food available actually exacerbated the negative effects of crowding, the results do not appear to be driven by nutritional deficits. The retarded growth rates and body sizes could have been induced by a build-up of toxic contaminates from fecal waste or from the stress of increasing the frequency of interaction with conspecifics (Peters and Barbosa 1977). Such stimuli could change an individual's developmental trajectory via effects on the neuroendocrine system (Hartfelder and Emlen 2005). Further supporting the possible role of nutrient-independent influences is the consistently stronger response of males for most of the traits examined. If a restricted diet were solely responsible, the larger bodied females should have been the sex with more pronounced developmental deficits given their need to provide more endogenous resources than the males.

Despite the pronounced changes observed in the traits measured, the stress of overcrowding the nymphs did not produce any effect on fluctuating asymmetry scores. A constant and low fluctuating asymmetry in the face of increasing environmental stress has been observed in other insects (David et al. 1998; Bjorksten et al. 2000; Mpho et al. 2000; Hoffmann et al. 2002) and may be indicative of a homeostatic mechanism that ensures the stable and coordinated development of paired traits, even if at the expense of overall size (Hoffmann & Woods 2001). It is possible that heightened asymmetries did occur as the nymphs were exposed to increasing stress, but these might have gone undetected by the use of simple linear trait measures. Hoffman et al. (2002) found that while environmental stressors did not produce fluctuating asymmetry changes in wing size, they did have a significant effect on the asymmetry of wing shape. Because different stressors can evoke different developmental responses, fluctuating asymmetry may still be a useful measure for *Lygus* exposed to challenges other than high density, as was found for *Culex pipiens* (Agnew et al. 2000; Mpho et al. 2000, 2002).

While most of the traits tracked in nymphs responded negatively to increased population density, their gonadal development did not. Both females and males exhibited increased gamete production as newly molted adults when exposed to overcrowding, although at the highest density these gains were negated. The increased density may trigger a competitive response among conspecifics to ensure that they can produce progeny earlier or in greater numbers to better compete for limited resources. The allocation of additional resources to reproductive development may in turn have contributed to their stunted somatic development. The decrease in gamete production in the transition from the 500 to 500DD density is likely the result of passing a threshold stress level after which the development of all traits becomes inhibited. The *Lygus* exposed to different population densities as adults were able to produce their first clutch of eggs at equivalent rates probably because they were able to rely on the endogenous reserves built up as nymphs reared under relatively hospitable conditions. Longer exposure to overcrowding might eventually produce a negative effect on fertility as the adults become increasingly reliant on newly acquired resources to produce eggs. It is also possible that individuals exposed to high densities at either stage of development may exhibit greatly reduced lifetime fitness (see Hooper et al. 2003), which would have gone undetected in this study. Nymphs in particular may suffer longer-lasting consequences given the pronounced effects on other aspects of their development. However, the stunted growth of these individuals is not necessarily indicative of a reduction in their fecundity because traits can be expressed independently (Karlsson and Wiklund 1984; Ohgushi 1996). Some adult insects are also able to compensate for developmental deficits incurred in earlier stages (Campero et al. 2008).

Just as the short-term reproductive responses to overcrowding may not be indicative of effects on lifetime fecundity, the heightened mortality rates observed in overcrowded nymphs and young adults may not reflect the longevity that the survivors can achieve. The weakest individuals are likely to be the first to die, leaving a more robust population (reviewed in Van Dongen 2006). Had more of the susceptible individuals survived, they would have probably exhibited greater developmental deficiencies, including higher fluctuating asymmetry scores, than the population that remained (Campero et al. 2008). Another possibility is that many of the dead nymphs were simply the victims of intraspecific predation (Beards and Leigh 1960; Khattat and Stewart 1977). Those that did survive to adulthood may exhibit equivalent longevities regardless of their experiences as nymphs.

The higher adult mortality rates over that of nymphs, despite a lack of other detrimental effects, may be due to differences in the way the two stages respond behaviorally to overcrowding. *Lygus* adults tend to be less aggregated than nymphs (Sevacherian and Stern 1972; Schotzko DJ and O'Keefe 1989) and will flee or defend themselves against unwanted contact with conspecifics. Being forced to endure a high contact rate may cause the adults to become agitated and aggressive to a greater extent than the nymphs. The added energy expenditure and potential for injury associated with increasingly agonistic interactions could compromise immune resistance and increase overall pathogen susceptibility (Peters and Barbosa 1977; Adamo 2006). A high population density might also enhance the rate of cannibalism in this omnivorous species, a common response in stressed or poorly sheltered populations of Mirids (Wheeler 2001). In addition to directly impacting mortality rates, cannibalism would also amplify the stressful nature of the rearing environment.

In conclusion, these results indicate that *L. hesperus* is quite sensitive to population density during its development. Although the long-term consequences on adult survivability and lifetime fecundity are unknown, there is sufficient evidence to caution against the experimental use of *Lygus* raised in crowded laboratory conditions. This study also provides a suite of responsive traits that can be used to effectively monitor other environmental stressors. These results can provide a guide by which future laboratory studies of this important pest species can be better designed and interpreted.

## Abbreviations

DD: double diet

## References

[bibr01] Adamo SA (2006). Comparative psychoneuroimmunology: Evidence from the insects.. *Behavioral and Cognitive Neuroscience Reviews*.

[bibr02] Agnew P, Haussy C, Michalakis Y (2000). Effects of density and larval competition on selected life history traits of *Culex pipiens quinquefasciatus* (Diptera: Culicidae).. *Journal of Medical Entomology*.

[bibr03] Agustı' N (1998). Biologia de *Dicyphus tamaninii* Wagner (Heteroptera: Miridae) i identificacio' molecular de les preses ingerides..

[bibr04] Allendorf FW, Leary RF, Soule' ME (1986). Heterozygosity and fitness in natural populations of animals.. *Conservation Biology. The Science of Scarcity and Diversity*.

[bibr05] Averill AL, Prokopy RJ (1987). Intraspecific competition in the tephritid fruit fly *Rhagoletis pomonella*.. *Ecology*.

[bibr06] Bancroft JS (2005). Dispersal and abundance of *Lygus hesperus* in field crops.. *Environmental Entomology*.

[bibr07] Beards GW, Leigh TF (1960). A laboratory rearing method for *Lygus hesperus* Knight.. *Journal of Economic Entomology*.

[bibr08] Bjorksten T, David P, Pomiankowski A, Fowler K (2000). Fluctuating asymmetry of sexual and nonsexual traits in stalk-eyed flies: A poor indicator of developmental stress and genetic quality.. *Journal of Evolutionary Biology*.

[bibr09] Campero M, De Block M, Ollevier F, Stoks R (2008). Metamorphosis offsets the link between larval stress, adult asymmetry and individual quality.. *Functional Ecology*.

[bibr10] Carrière Y, Ellsworth PC, Dutilleul P, Ellers-Kirk C, Barkley V, Antilla L (2006). A GIS-based approach for area wide pest management: The scales of *Lygus hesperus* movements to cotton from alfalfa, weeds, and cotton.. *Entomologia Experimentalis et Applicata*.

[bibr11] Castaňé C, Alomar Ó, Riudavets J, Gemeno C (2007). Reproductive biology of the predator *Macrolophus caliginosus*: Effect of age on sexual maturation and mating.. *Biological Control*.

[bibr12] Clarke GM, McKenzie LJ (1992). Fluctuating asymmetry as a quality control indicator for insect mass rearing processes.. *Journal of Economic Entomology*.

[bibr13] David P, Hingle A, Greig D, Rutherford A, Pomiankowski A, Fowler K (1998). Male sexual ornament size but not asymmetry reflects condition in stalk eyed flies.. *Proceedings of the Royal Society London* B.

[bibr14] Debolt JW (1982). Meridic diet for rearing successive generations of *Lygus hesperus*.. *Annals of the Entomological Society of America*.

[bibr15] Demirel N, Cranshaw W (2006). Surveys of *Lygus* spp. and their movement on cultivated crops and non-cultivated habitats throughout growing season in Colorado.. *Pakistan Journal of Biological Sciences*.

[bibr16] Hartfelder K, Emlen DJ, Gilbert LI, Iatrou K, Gill SS, 3 (2005). Endocrine control of insect polyphenism.. *Comprehensive Molecular Insect Science*.

[bibr17] Hoffmann AA, Woods RE (2001). Trait variability and stress: Canalization, developmental stability and the need for a broad approach.. *Ecology Letters*.

[bibr18] Hoffmann AA, Collins E, Woods R (2002). Wing shape and wing size changes as indicators of environmental stress in *Helicoverpa punctigera* (Lepidoptera: Noctuidae) moths: Comparing shifts in means, variances, and asymmetries.. *Environmental Entomology*.

[bibr19] Hooper HL, Sibly RM, Hutchinson TH, Maund SJ (2003). The influence of larval density, food availability and habitat longevity on the life history and population growth rate of the midge *Chironomus riparius*.. *Oikos*.

[bibr20] Jackson CG, Debolt JW, Ellington J, Nechols JR, Andres LA, Beardsley JW, Goeden RD, Jackson CG (1995). *Lygus* bugs.. *Biological Control in the Western United States*.

[bibr21] Karlsson B, Wiklund C (1984). Egg weight variation and lack of correlation between egg weight and offspring fitness in the wall brown butterfly *Lusiommata megem*.. *Oikos*.

[bibr22] Khattat AR, Stewart RK (1977). Development and survival of *Lygus lineolaris* exposed to different laboratory rearing conditions.. *Annals of the Entomological Society of America*.

[bibr23] Leung B, Forbes MR (1996). Fluctuating asymmetry in relation to stress and fitness: Effects of trait type as revealed by meta-analysis.. *Ecoscience*.

[bibr24] Leung B, Forbes MR, Houle D (2000). Fluctuating asymmetry as a bioindicator of stress: Comparing efficacy of analyses involving multiple traits.. *American Naturalist*.

[bibr25] Ma WK, Ramaswamy SB (1987). Histological changes during ovarian maturation in the tarnished plant bug *Lygus lineolaris* (Palisot de Beauvois) (Hemiptera: Miridae).. *International Journal of Insect Morphology and Embryology*.

[bibr26] Mpho M, Callaghan A, Holloway GJ (2002). Temperature and genotypic effects on life history and fluctuating asymmetry in a field strain of *Culex pipiens*.. *Heredity*.

[bibr27] Mpho M, Holloway GJ, Callaghan A (2000). The effect of larval density on life history and wing asymmetry in the mosquito *Culex pipiens*.. *Bulletin of Entomological Research*.

[bibr28] Ohgushi T (1996). Consequences of adult size for survival and reproductive performance in a herbivorous ladybird beetle.. *Ecological Entomology*.

[bibr29] Palmer RA, Strobeck C (1986). Fluctuating asymmetry: Measurement, analysis, patterns.. *Annual Review of Ecology and Systematics*.

[bibr30] Patana R (1982). Disposable diet packet for feeding and oviposition of *Lygus hesperus* (Hemiptera: Miridae).. *Journal of Economic Entomology*.

[bibr31] Peters TM, Barbosa P (1977). Influence of population density on size, fecundity, and development rate of insects in culture.. *Annual Review of Entomology*.

[bibr32] Reiskind MH, Walton ET, Wilson ML (2004). Nutrient-dependent reduced growth and survival of larval *Culex restuans* (Diptera: Culicidae): Laboratory and field experiments in Michigan.. *Journal of Medical Entomology*.

[bibr33] Rhainds M, English-Loeb G (2003). Testing the resource concentration hypothesis with tarnished plant bug on strawberry: Density of hosts and patch size influence the interaction between abundance of nymphs and incidence of damage.. *Ecological Entomology*.

[bibr34] Schotzko DJ, O'Keefe LE (1989a). *Lygus hesperus* distribution and sampling procedures in lentils.. *Environmental Entomology*.

[bibr35] Schotzko DJ, O'Keefe LE (1989b). Geostatistical description of the spatial distribution of *Lygus hesperus* (Heteroptera: Miridae) in lentils.. *Journal of Economic Entomology*.

[bibr36] Sevacherian V, Stern VM (1972). Spatial distribution patterns of *Lygus* bugs in California cotton fields.. *Environmental Entomology*.

[bibr37] Simpson SJ, McCaffery AR, Hägele BE (1999). A behavioural analysis of phase change in the desert locust.. *Biological Reviews of the Cambridge Philosophical Society*.

[bibr38] Strong FE, Sheldahl JA, Hughes PR, Hussein EMK (1970). Reproductive biology of *Lygus hesperus* Knight.. *Hilgardia*.

[bibr39] Van Dongen S (2006). Fluctuating asymmetry and developmental instability in evolutionary biology: Past, present and future.. *Journal of Evolutionary Biology*.

[bibr40] Wheeler AG (2001). *Biology of the Plant Bugs (Hemiptera: Miridae).*.

